# Molecular diversity and biennial circulation of enterovirus D68: a systematic screening study in Lyon, France, 2010 to 2016

**DOI:** 10.2807/1560-7917.ES.2018.23.37.1700711

**Published:** 2018-09-13

**Authors:** Rolf Kramer, Marina Sabatier, Thierry Wirth, Maxime Pichon, Bruno Lina, Isabelle Schuffenecker, Laurence Josset

**Affiliations:** 1 Centre National de Référence des Enterovirus et Parechovirus, Laboratoire de Virologie, Institut des Agents Infectieux, HCL, Hôpital de la Croix-Rousse, Lyon, France; 2 European Public Health Microbiology Training Programme (EUPHEM), European Centre for Disease Prevention and Control, Stockholm, Sweden; 3 These authors contributed equally; 4 Laboratoire Biologie Intégrative des Populations, Evolution Moléculaire, EPHE, PSL University, Paris, France; 5 Institut Systématique Evolution Biodiversité (ISYEB), EPHE, MNHN, CNRS, Sorbonne Université, Paris, France; 6 Virpath, CIRI, Université de Lyon, INSERM U1111, CNRS 5308, ENS de Lyon, UCBL, Lyon, France

**Keywords:** enteroviruses, EV-D68, respiratory infections, laboratory surveillance, clinic, typing

## Abstract

Understanding enterovirus D68 (EV-D68) circulation patterns as well as risk factors for severe respiratory and neurological illness is important for developing preventive strategies. **Methods**: Between 2010 and 2016, 11,132 respiratory specimens from hospitalised patients in Lyon, France, were screened for EV-D68 by PCR. Phylogenetic relationships of the viral-protein-1 sequences were reconstructed using maximum-likelihood and Bayesian-Markov-Chain-Monte-Carlo approaches. **Results:** Overall, 171 infections with a biennial pattern were detected, including seven, one, 55, none, 42, one and 65 cases annually during 2010–16. Children (< 16 years-old; n = 150) were mostly affected and 71% (n = 121) of the total patients were under 5 years-old. In 146 patients with medical reviews, 73% (n = 107) presented with acute respiratory distress. Among paediatric patients with medical reviews (n = 133), 55% (n=73) had an asthma/wheezing history, while among adults (n = 13), 11 had underlying diseases. In total, 45 patients had severe infections and 28 patients needed intensive care unit stays. No acute flaccid myelitis (AFM) was detected. We found genotypes A, B1, B2 B3 and D circulating, and no associations between these and clinical presentations. During the study, new genotypes continuously emerged, being replaced over time. We estimated that ancestors of currently circulating genotypes emerged in the late-1990s to 2010. Rises of the EV-D68 effective population size in Lyon coincided with infection upsurges. Phylogenetic analyses showed ongoing diversification of EV-D68 worldwide, coinciding with more infections in recent years and increases of reported AFM paediatric cases. **Conclusions:** Reinforcement of diagnostic capacities and clinical-based surveillance of EV-D68 infections is needed in Europe to assess the EV-D68 burden.

## Introduction

Enterovirus D68 (EV-D68) is a re-emerging pathogen which was first isolated in 1962 from children with pneumonia and bronchiolitis [1]. Until 2008, EV-D68 was rarely reported and represented only ca 0.1% of all clinical enterovirus isolates in the United States (US) [2]. Between 2008 and 2014, small outbreaks of EV-D68 respiratory disease were observed worldwide [3]. In 2014, the first large outbreak of EV-D68 was reported in North America associated with considerable morbidity and mortality [4]. A total of 1,152 cases, mostly paediatric patients, were reported between August and December in 49 US states. Clinical manifestations were mainly respiratory distress, tachypnea, hypoxaemia, wheeze, and chest pain. A history of asthma and wheezing was commonly observed. During this outbreak, ca 40% of respiratory samples from patients with severe respiratory symptoms tested by the nation's health protection agency were positive for EV-D68 [5]. Notably, an upsurge of polio-like neurological symptoms, e.g. acute flaccid myelitis (AFM), coincided with the EV-D68 outbreak and AFM patients were observed to have a 10-fold increased chance being EV-D68 positive [6]. Consequently, surveillance studies for EV-D68 were launched worldwide which resulted into increased detection and enhanced data collection. In Europe, local clusters of EV-D68 infections were repeatedly reported in 2014 and 2016, including cases of severe respiratory disease and AFM [7-12]. Longitudinal surveillance data are hardly available and information on circulation of EV-D68 remains limited, although endemic circulation appears to exist worldwide: (i) an Australian study detected 55 cases from 2007 to 2017 with peaks of infections in 2011 and 2013 for the July to October periods [13], (ii) a Taiwanese study detected EV-D68 from 2007 to 2014 without clear pattern [14], (iii) a study from Colorado reported a biennial pattern from 2014 to 2016 with 117 cases being intensive care patients [15] and (iv) a Dutch study identified 27 cases with a biennial pattern through a combined sentinel influenza-like illness/acute respiratory infections surveillance and enterovirus surveillance between 2011 and 2014 [16].

Understanding circulation patterns as well as identification of risk factors for severe respiratory and neurological illness is essential to identify outbreaks and develop preventive strategies. Previous molecular studies suggest that EV-D68 has undergone a rapid evolution since the mid-1990s. Genetic diversification led to the emergence of three genotypes (clades A to C) and multiple sub-lineages. However, it remains to be determined if emergence of new clades is linked with increased susceptibility of the population or with the severity of disease [9,12].

We conducted a systematic, longitudinal screening study of EV-D68 over a period of 7 years. We analysed circulation patterns, clinical manifestations, patient characteristics, phylogenetic relationships and genetic diversity of the virus. Putting our results in context, we infer objectives for future surveillance of EV-D68 infections.

## Methods

### Patient selection and specimen screening

We screened all available respiratory specimens collected from patients hospitalised or admitted to emergency units in Lyon University hospital (paediatric patients < 16 years of age; adults ≥ 16 years of age). Long-term hospitalised patients in paediatric haematology and gastroenterology units were excluded from the study to consider only EV-D68 community cases.

Systematic screening of EV-D68 was performed using EV-D68 real-time PCR on all available respiratory specimens detected positive for enterovirus/human rhinovirus (EV/HRV) during the summer/autumn period in 2010 to 2016 (Table). Briefly, after the 2014 alert, we started investigating virus circulation in 2014 between July and December. Subsequently, we screened retrospectively samples collected in 2010 to 2013 between August/September and November (corresponding to peak prevalence of EV-D68 in 2014). Prospective screening was conducted in 2015 and 2016. While screening in 2015 was conducted as in the 2010 to 2013 years, the screening period in 2016 was extended (May to December) because of the early detection of EV-D68 through routine genotyping of respiratory specimens by the French enterovirus laboratory network and the reporting of an increase of severe neurological cases in the capital of Sweden (Stockholm), as well as in France and in the Netherlands [8-10].

**Table:** Sample collection and demographics of enterovirus D68 (EV-D68)-infected patients, Lyon, France, 2010–2016

Year	Screening period (week)	Samples^a^ tested (total)	Number of EV/HRV-positive samples and proportion among samples tested		Number of EV-D68-positive samples and proportion among samples tested		Number and proportions of patients with EV-D68 by age group among those tested positive									
							< 1y		1–4y		5–15y		16–64y		≥ 65y	
			n	%	N	%	n	%	n	%	n	%	n	%	n	%
2010	37–48	649	304	46.8	7	1.1	1	14.3	2	28.6	3	42.9	1	14.3	0	0.0
2011	37–48	1,075	284	26.4	1	0.1	0	0.0	1	100	0	0.0	0	0.0	0	0.0
2012	32–48	1,442	384	26.6	55	3.8	16	29.1	24	43.6	9	16.4	5	9.1	1	1.8
2013	37–48	1,133	347	30.6	0	0.0	0	0.0	0	0.0	0	0.0	0	0.0	0	0.0
2014	27–52	2,478	492	19.9	42	1.7	13	31.0	13	31.0	7	16.7	8	19.1	1	2.4
2015	37–53	2,109	401	19.0	1	0.1	0	0.0	1	100	0	0.0	0	0.0	0	0.0
2016	22–48	2,246	557	24.8	65	2.9	17	26.2	33	50.8	10	15.4	4	6.2	1	1.5

EV: enterovirus; HRV: human rhinovirus; n: absolute numbers; week: calendar week; y: years old.

^a^ Respiratory samples collected from patients originating from the community, who were hospitalised or admitted to emergency units in Lyon University hospital.

### Enterovirus-D68 detection

RNA extraction was carried out using NucliSENS easyMAG (BioMérieux, France). Specimens were systematically tested for EV/HRV by real-time reverse transcription (RT)-PCR (Rhino and EV/Cc r-gene, BioMérieux, France). For detection of EV-D68, real-time RT-PCR was performed on ABI 7500 thermocycler (Applied Biosystem, Life Technologies California, US) using primers and protocols described by Poelman et al. [17].

### Molecular typing of enterovirus-D68

Complete viral-protein-1 (VP1) region sequences of EV-D68 positive samples were amplified with EV-D68-specific in-house primers and sequenced using the Sanger method [12]. If a complete *VP1* sequence could not be obtained, a partial *VP1* sequence was amplified using the method by Nix et al. [18]. Sequences were analysed using DNASTAR Lasergene 8 SeqMan (Madison, Wisconsin, US). GenBank accession numbers of sequences collected in Lyon from 2010 to 2016 are KP196362–72, KP196374–76, KP307992, KP406467–73, KT220441–51, KY272868–920, KY272922–60.

### Clinical presentations

A review of medical charts was carried out retrospectively providing information on: age and sex; clinical presentations including fever (threshold: > 38.5 °C), cough, rhinitis, pharyngitis, bronchitis or bronchiolitis, acute respiratory distress, pneumonia; severity criteria at admission such as need for intensive care and/or need for oxygen; length of hospitalisation; final diagnosis; presence or absence of underlying asthma or wheezing, atopy, and chronic respiratory disease. Informed consent was not required for this study.

### Sequence alignments

**Figure 1:**
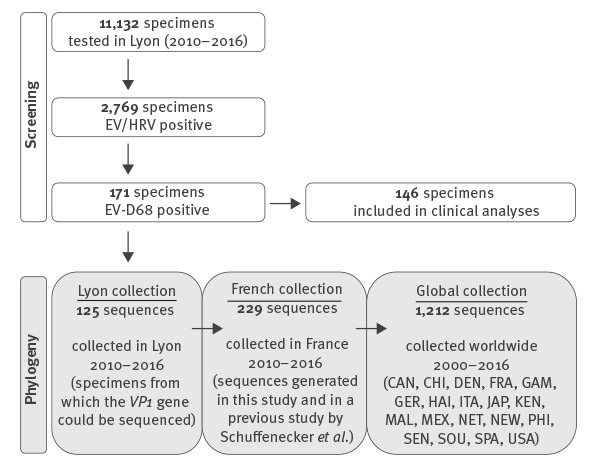
Screening for enterovirus-D68 (EV-D68)-positive samples in Lyon, and selection of subsets for clinical analyses, or for sequencing and phylogenetic analyses together with French or global EV-D68 sequence collections, 2010–2016

CAN: Canada; CHI: China; DEN: Denmark; EV-D68: enterovirus D68; HRV: human rhinovirus; FRA: France; GAM: The Gambia; GER: Germany; HAI: Haiti; ITA: Italy; JAP: Japan; KEN: Kenya; MAL: Malaysia; MEX: Mexico; NCBI: National Center for Biotechnology Information; NET: Netherlands; NEW: New Zealand; PHI: Philippines; SEN: Senegal; SOU: South Africa; SPA: Spain; USA: United States.

Three different sets of EV-D68 VP1 sequences were aligned using MAFFT 7 [19], namely sequences from Lyon, as well as sequences from French and worldwide datasets (Figure 1).

White boxes show total numbers of respiratory specimens included in each screening step. Of 171 patients infected with EV-D68, 25 were excluded from clinical analyses because another microorganism or health condition was likely to be responsible for the clinical symptoms. Grey boxes show total numbers of sequences included in each dataset for phylogenetic analyses. For the Lyon dataset, viral-protein-1 (VP1) gene sequences could be obtained from 125 of 171 specimens, e.g. 6, 1, 42, 0, 33, 0 and 43 for each respective year from 2010 to 2016. The Lyon *VP1* sequence dataset was included in a French dataset, which comprised other sequences collected in France in 2014 and published by Schuffenecker et al. [12]. The French dataset was in turn included in a global dataset with representative sequences gathered from NCBI.

### Phylogenetic inferences and coalescent analyses

A transitional substitution model with gamma distributed rate heterogeneity and proportion of invariable sites were selected based on Akaike’s information criterion using jModelTest [20]. Phylogenetic relationships were reconstructed with a maximum likelihood (ML) approach implemented in PhyML 3.412 under the above selected model and W-IQ-TREE for the global dataset [21,22]. Robustness of ML tree topology was assessed with bootstrapping analyses of 1,000 pseudo-replicated datasets. Root-to-tip genetic distance inferred from ML trees were regressed against time of sampling (years) using the TEMPEST 1.5 programme [23]. The specific rate of evolution for EV-D68 was estimated from ‘serially-sampled’ viruses with known sampling dates. Evolutionary rates were obtained using the Bayesian Markov-Chain-Monte–Carlo (MCMC) approach implemented in Bayesian-Evolutionary-Analysis-by-Sampling-Trees (BEAST) software 1.8.4 [24]. An uncorrelated lognormal relaxed molecular clock was chosen. Evolutionary rates and tree topologies were analysed using General-Time-Reversible (GTR) and Hasegawa-Kishino-Yano (HKY) substitution models with gamma distributed among-site rate variation with four rate categories. Constant-sized, logistic, exponentially growing coalescent models were used. A bayesian skyline plot model was considered, based on a general, non-parametric prior that enforces no particular demographic history. We used piecewise-linear-skyline model with 20 groups and compared marginal likelihoods for each model using Bayes factors estimated in TRACER 1.6. Bayes factors represent the ratio of the marginal likelihood of compared models. For each analysis, two independent runs of 200 million steps were performed with trees being sampled every 20,000 steps, the first 1,000 trees were discarded. We summarised MCMC samples using maximum-clade-credibility topology found with TREEANNOTATOR 1.5 (branch lengths in years). Bayesian skyline plot was reconstructed using the posterior tree sample and TRACER 1.6 to show effective population size which translates census sizes of a real population into the size of an idealised population showing the same rate of loss of genetic diversity as the real population under study. This mathematical estimate of the population size correlates with the relative genetic diversity and allows reconstructing pathogen population size fluctuations through time and therefore captures demographic expansions or sharp decreases. Statistical confidence in parameter estimates was represented by values for the 95% highest-probability-density intervals around the marginal posterior parameter means.

### Statistics

One-way analysis-of-variance was used to test for significant differences in age contribution between epidemic years. We used Fisher's exact test to test for associations between genotypes and clinical presentations. A significant difference was defined as a p value < 0.05.

## Results

### Enterovirus-D68 circulation

**Figure 2:**
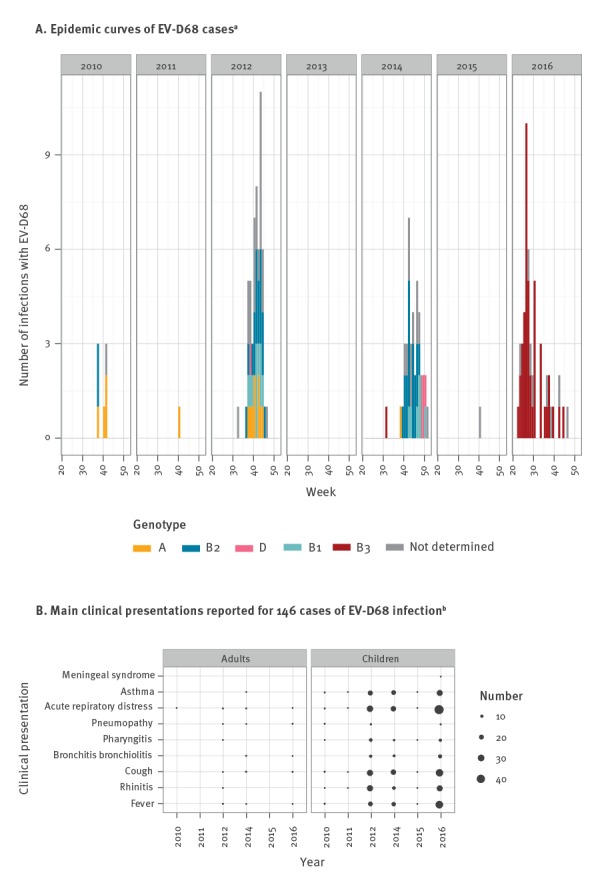
A. Distribution of enterovirus D68 (EV-D68) infections (n = 171) and B. Clinical presentation of a subset of infected patients (n = 146), Lyon, France, 2010–2016

In total, 11,132 specimens were screened, of which 52.9% (n = 5,886) were collected from paediatric patients (Figure 1). Types of specimens consisted mostly of nasopharyngeal aspirates (58.5%; n = 6,511) and nasopharyngeal swabs (28%; n = 3,117). EV/HRV were detected in 24.9% of specimens (n = 2,769), of which 6.2% (n = 171) were positive for EV-D68 (1.5% of all specimens). A total of 171 EV-D68 infections were detected between 2010 and 2016. Figure 2A shows weekly and yearly distribution of EV-D68 cases. A biennial pattern was observed; in 2010: seven cases (2.3% of EV/HRV positive samples), in 2011: one case (0.4%), in 2012: 55 cases (14.3%), in 2013: no case, in 2014: 42 cases (8.5%), in 2015: one case (0.3%), in 2016: 65 cases (11.7%; Table). Years with case numbers > 1 (2010, 2012, 2014 and 2016) are referred to as epidemic years. Peaks of infections were observed during autumn in 2012 and 2014 but the peak shifted to the summer period in 2016 (2012: week 43; 2014: week 46; 2016: week 26).

EV-D68: enterovirus 68.

^a^ Case numbers (i.e. numbers of EV-D68 infections) are shown for every week of the screening periods. Different colours represent different genotypes of EV-D68.

^b^ Of 171 patients infected with EV-D68, 25 were excluded from clinical analyses because another microorganism or health condition was likely to be responsible for the clinical symptoms, hence clinical presentation is only shown for 146 patients. Adult and paediatric patients (< 16 years of age) are presented separately. The sizes of black bubbles are proportional to case numbers for each diagnosis. Multiple clinical presentations may be reported for one patient.

### Demographics of enterovirus-D68 positive patients

Both children and adults were affected by EV-D68 without sex predilection in the study period (female to male ratio 1:1.3). Of 171 infected patients, 150 (87.7%) were children (Table), 121 patients (70.8%) were under 5 years and 47 (27.5%) were below 1 year of age. Three patients (1.8%) were 65 years or older. Excluding 2010 due to low case numbers, proportions of patients in different age groups were relatively stable in different epidemic years (Table), e.g. proportions of patients < 5 years were 72.7%, 61.9% and 76.9% respectively. No significant differences in sex ratio nor in age at diagnosis were found between epidemic years 2012 to 2016 (p = 0.19).

### Clinical presentations

Of the 171 patients, another microorganism or health condition was likely to be responsible for clinical symptoms for 25, so these were excluded from analyses on clinical presentation. We reviewed medical charts of the remaining 146 patients (133 children and 13 adults). A history of asthma or wheezing was known for 73 paediatric patients (54.9%) and two adults. Underlying respiratory diseases (n = 10), including chronic obstructive pulmonary disease (COPD) and cystic fibrosis, or recent history of tissue transplantation (n = 1) were known for 11 of the 13 EV-D68 infected adults. Overall, 95.9% (n = 140) had respiratory symptoms (Figure 2B). Acute respiratory distress was reported in 101 of the 133 children (75.9%) and six of the 13 adults. Asthma was diagnosed in 53.4% of children (71/133) but was rarely observed in adults (n = 1). Bronchitis/bronchiolitis was reported for 33 of 133 children and for three of 13 adults. During epidemic years 2012, 2014 and 2016, asthma and bronchitis/bronchiolitis were reported at constant levels. No significant associations were found between clinical presentations and genotypes of the infecting virus (p > 0.05). In total, 40 children (30.1%) and five adults (38.5%) were considered having severe infections. The average length of hospital stays fell in the range of 3.2 to 4.4 days in epidemic years. On average, adults had significantly longer stays than children (7.2 days vs 3.3 days; p < 0.001). Intensive care unit stays were reported for 28 patients, 24 of those were children. On average, adults stayed twice as long as paediatric patients (8 days vs 3.9 days) but the difference was not statistically significant. No AFM case was observed in association with EV-D68 infections. Except for one child (< 1 year of age) who died of myocarditis, outcomes were favourable.

### Phylogeny

**Figure 3:**
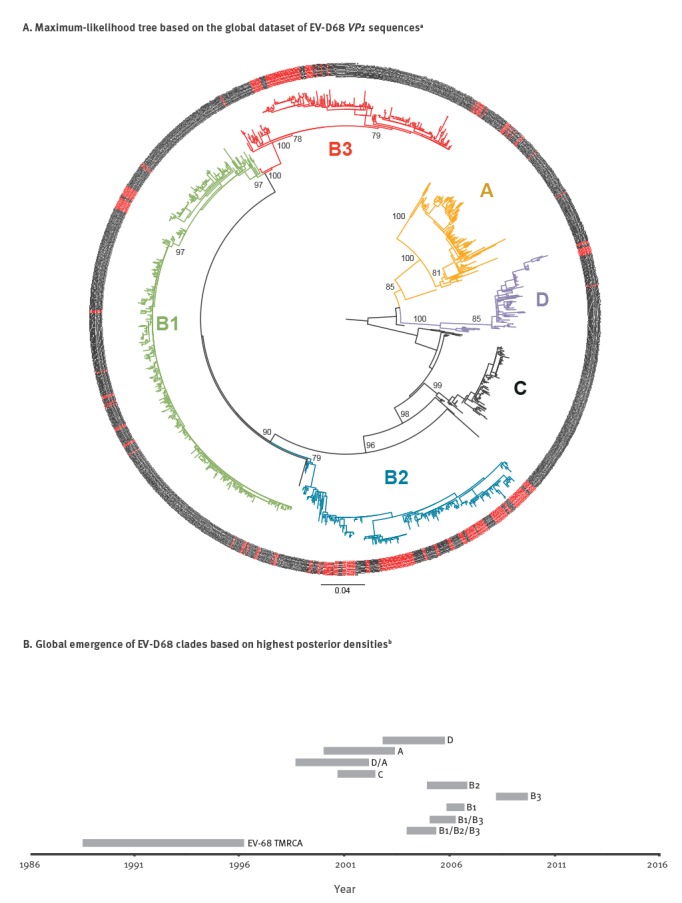
A. Phylogenetic analyses and B. Date of emergence of enterovirus D68 clades based on a worldwide viral-protein-1 (VP1) gene sequence dataset, 2000–2016 (n = 1,212 sequences)

GTR: general-time-reversible; TMRCA: the most recent common ancestor.

^a^ Phylogeny was generated with IQ tree under the GTR + F + R4 evolutionary model (highest likelihood) and mid-point rooting. Numbers presented on main branches correspond to bootstrap values. On the tree leaves (outer circle), samples collected in France are highlighted in red. The scale shows the length of branch representing 0.04 nt substitutions per site.

^b^ The bars represent the time range in which TMRCAs of the respective clades first emerged. TMRCA of all currently circulating viruses dates back to between 1989 and 1996.

Since recombination events can dramatically distort and affect tree topologies, we first tested our datasets for recombination using pairwise-homoplasy-index test [25]. No recombination event was evidenced from this analysis (p > 0.3). To understand the dynamic of the EV-D68 population, we analysed sequences from Lyon in the context of other sequences collected in France during the study period (French dataset) as well as in the context of samples collected worldwide (worldwide dataset). Based on the maximum likelihood tree of the *VP1* sequences, the global topology and diversity of EV-D68 was concordant with clades defined by Tokarz et al. and Gong et al. (Figure 3A) [3,26]. Nearly all clades except clade C were represented in France during the study period (A, B1, B2, B3 and D).

**Figure 4:**
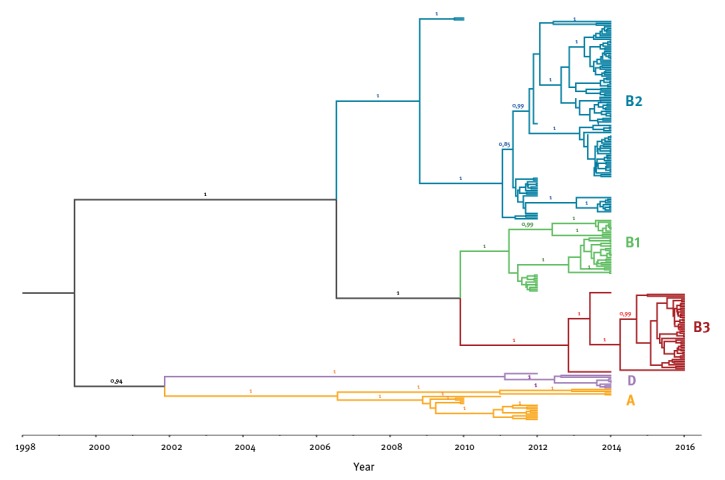
Time tree based on Bayesian Markov-Chain-Monte-Carlo analysis of the French viral-protein-1 gene sequence dataset of enterovirus D68, 2010–2016

For the French dataset, we additionally generated a Bayesian time-tree which confirmed the phylogenetic topology (Figure 4). The estimated short-term mutation rate corresponded to 5.54 x 10–3 substitutions per nt site per year, similar to the value reported previously in Tokarz et al. on a worldwide dataset [3]. As shown on Figure 2A (Lyon dataset) and Figure 4 (French dataset), new clades and sub-clades were emerging continuously and older clades were getting replaced over time. In 2012, three clades (A, B1 and B2) were co-circulating, of which A and B2 were already detected in 2010. In 2014, clade B2 was the most predominant genotype. Finally, all isolates collected in 2016 belonged to clade B3 which was first detected in 2014. Clade D represented a less frequently detected genotype, which was first detected in 2012.

Different genotypes are colour-coded and clades are shown.

On the worldwide dataset, we estimated the time to the most recent common ancestor (TMRCA) of all currently circulating EV-D68 viruses. The TMRCA dated back to 1989 to 1996 as indicated in Figure 3B. From this TMRCA, ancestors of all currently circulating clades evolved within a decade from late-1990s to 2010. Ancestors of specific clades appeared in the following order: C, A, then D, followed by B2, B1 and B3.

**Figure 5:**
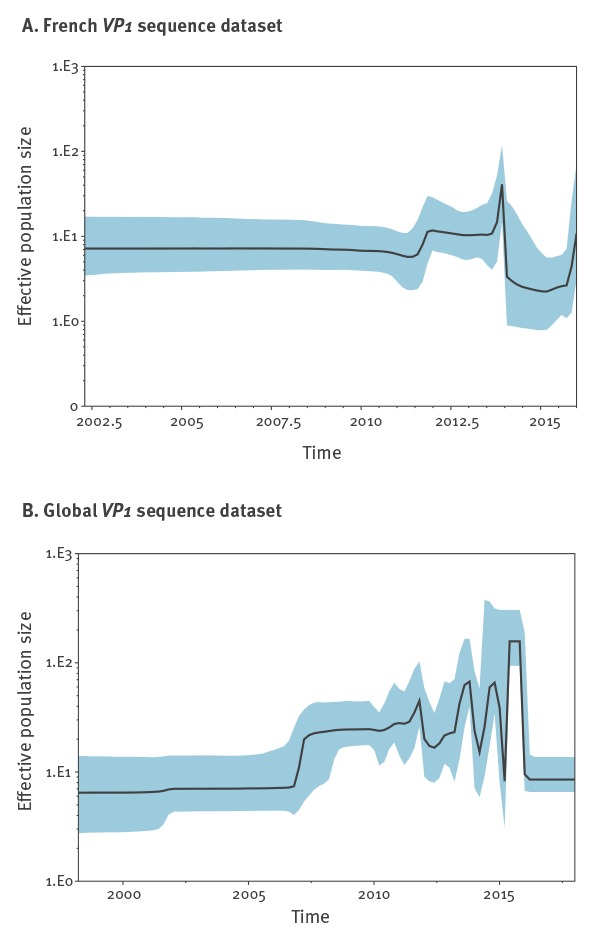
Bayesian skyline plots showing the effective population size fluctuations of enterovirus D68 in the French and global viral-protein-1 (VP1) gene sequence datasets, 2010–2016

We tested the performance of various demographic models which favoured a Bayesian skyline model with a relaxed molecular clock (data not shown). During our study period, rises of the EV-D68 effective population size in the French dataset were observed to occur biennially, namely in 2012, 2014 and 2016 (Figure 5A) and coincided with epidemic years of EV-D68 in Lyon. Likewise, the sharp decline after 2014 and the sudden rise for 2016 coincided with replacement of clades detected in 2014 (and before) by a new clade B3. At a global scale, we observed regular oscillations in the effective population size of EV-D68. However, no biennial cycle was observed for the global EV-D68 population (Figure 5B).

The black line is the median estimate of the estimated effective population size. The coloured shade represents the upper and the lower estimates of 95% interval. Due to formatting restrictions, time on x-axis is displayed in full years (e.g. 2015) and half years (e.g. 2002.5).

## Discussion

This is the first longitudinal study which systematically screened adult and paediatric patients hospitalised or admitted to the emergency unit of a French tertiary hospital during a 7 year period. We detected 171 EV-D68 infections comprising all age groups. Our data confirmed a strong local biennial pattern in EV-D68 circulation which was similarly reported elsewhere in Europe [27,28]. Epidemic curves suggested that the peak of activity was detected during epidemic years. The outbreaks respectively peaked after week 40 in autumn of 2012 and 2014, but earlier (week 27) in summer 2016. Together with consecutive detection from late spring to winter (week 22 to 51) our data suggest that circulation depends on variable factors, such as genotype, climate and base-line immunity of the population. This makes prediction of outbreaks more difficult. The reasons for the two-year interval remain to be elucidated but population size of susceptible individuals and fitness of emerging viral genotypes are likely to be major factors among other unknown variables. Similar observations were also made for other respiratory viruses and enteroviruses, including EV-A71 in Malaysia [29].

Nearly three quarters of the patients were children under 5 years-old, probably because they are more likely to be hospitalised than older patients. Infants (< 1 year-old) were particularly vulnerable (prone to hospitalisation). EV-D68 infections leading to hospitalisation decreased with age. Similar observations were made for other enteroviruses, such as EV-A71 [2,29]. This might be explained by small airways of young children which makes them prone to develop severe symptoms and more likely to be hospitalised. Noteworthy, hospital stays were longer for adults than for children but most hospitalised adults had underlying respiratory tract conditions like COPD, cystic fibrosis or were treated for an organ transplantation which assumingly made them prone to develop severe symptoms due to EV-D68 infection. Those underlying health conditions may increase vulnerability and represent a risk factor for adults to require hospitalisation when infected with EV-D68. For children, asthma or wheezing was known for about half of the hospitalisations and represents a potential risk factor which was similarly reported in previous outbreaks [5].

In contrast to other enteroviruses, EV-D68 is known to be primarily associated with respiratory symptoms [30,31]. Observed clinical presentations were concurrent with symptoms described in the case definition used in the Children’s Mercy Hospitals during the US outbreak in 2014 [5]. Adults required longer hospital stays for recovery than children. Benign symptoms such as rhinitis as well as pharyngitis may have been under-reported. Longer hospital stays for adults may be attributed to the potential risk factors described above rather than being representative for the general adult population without underlying health conditions. Similar diagnoses were observed in every year of the study period and different clades were not observed to be associated with clinical presentations and severity. However, since whole genome sequencing (WGS) was not performed, mutations linked with clinical presentations in genes other than *VP1* cannot be excluded. 

Although no case of AFM was reported in Lyon during the study period, five cases of AFM with EV-D68 infection (one in 2014 and four in 2016) were reported in France and cases of myelitis associated with EV-D68 infection have now been detected in 14 countries on six continents [8]. Epidemiological and animal model data indeed support an association between EV-D68 and AFM as well as a common physiopathology between EV-D68 and poliovirus [6,8,9,32,33]. This has to be taken into account in public health policies and raises the necessity to reinforce both AFM surveillance and EV-D68 diagnostics in Europe.

To compare the circulation pattern with genetic variability of the virus, we analysed our data in the context of EV-D68 sequences collected elsewhere. We observed a strong ongoing diversification of the virus as demonstrated on different levels: (i) newly emerging clades in epidemic years in Lyon, (ii) rises of effective population size of the virus in France correlated with epidemic years in Lyon, (iii) dynamic effective population size of EV-D68 globally and (iv) evolution of all ancestors of currently circulating genotypes within a decade. Previous studies had concluded that diversification of EV-D68 within different clades was a recent phenomenon and got amplified in mid-1990s [3]. Our analyses confirmed this. Notably, our analyses also confirmed the existence of the recently described clade D [26]. Diversification of EV-D68 increased in the late-1990s/beginning-2000s with the appearance of common ancestors for clade A, C and D. Another major diversification took place around mid-2000s with the appearance of common ancestors of B2, B1 and B3. The order of appearances shows a similar pattern than the order of detection of specific clades in Lyon from 2010 to 2016. Clade A and B2 were detected in 2010, B1 was first detected in 2012 and B3 was first detected in 2014. Additionally, Clade C was observed in France in 2008 before the study period (data not shown). Overall, we observed continuous emergence and replacement of clades in epidemic years in Lyon. In 2016, clade B3 was observed to be sole genotype in Lyon and other parts of France (data not shown). Other European countries reported outbreaks with the same genotype [9,10]. We also observed a coinciding elevation of the effective population size in France and upsurge of infections in our screening period. Such strong congruence between the genetic diversity and (local) epidemiological data was similarly observed for human influenza A virus and EV-A71 [29,34,35]. Authors of these studies offer a possible explanation which might likewise apply to the circulation of EV-D68: the virus persists in the population between epidemics with a strong selection pressure on fitness of the virus which results in a waning immunity of the population over time. Additionally, the susceptible population size increases by naïve newborns. We observed that young children, particularly newborns, are most vulnerable to infection and new clades of the virus emerged continuously. We hypothesise that EV-D68 epidemics occur when the susceptible population is large enough for the virus to spread. In Lyon, this threshold was reached every 2 years during the study period. On the global scale, fluctuations in genetic diversity were even more drastic: A perfect biennial pattern could not be observed which is probably due to different climatic and demographic environments worldwide which favour EV-D68 epidemics in different cycles. However, oscillating ongoing genetic dynamics of the global EV-D68 population is in evidence. For other enteroviruses, indicators for heterogeneity in pathogenicity of different genotypes were likewise observed when comparing circulating viruses isolated from sewage with clinical isolates from the same community [36]. Increased pathogenicity in a given EV-D68 virus would favour its positive selection.

Considering the recent rapid evolution and increase in effective population size of EV-D68, frequent and large outbreaks are likely to occur in the future. This and the severe neurological complications observed in some EV-D68 cases justify the need for continuous surveillance of EV-D68 infections. On the basis of our and other studies, routine diagnostics for respiratory enteroviruses and a sentinel clinical surveillance of EV-D68 infections in paediatric hospitals should focus on children under 5 years of age. This should include enterovirus-typing in respiratory samples and subsequent WGS of EV-D68. WGS-based identification of possible genetic factors or mutations associated with disease severity can be used for refining case definition in outbreaks of EV-D68, e.g. indicators for genetic factors associated with neurological manifestations were found retrospectively for the US outbreak 2014 [37]. Surveillance using WGS will become more powerful with growing sequence databases as well as integrated epidemiological and clinical information. Monitoring virus diversification and rises in genetic diversity in such manner would facilitate identification of reasons as well as driving forces of ongoing genetic dynamics in the EV-D68 population.
